# Simulation study on the mining conditions of dissolution of low grade solid potash ore in Qarhan Salt Lake

**DOI:** 10.1038/s41598-021-88818-z

**Published:** 2021-05-18

**Authors:** Ruiqin Li, Chenglin Liu, Pengcheng Jiao, Yufei Hu, Wanping Liu, Shijun Wang

**Affiliations:** 1 MNR Key Laboratory of Metallogeny and Mineral Assessment, Institute of Mineral Resources, Chinese Academy of Geological Sciences, Beijing, 100037 China; 2 School of Earth and Space Sciences, Peking University, Beijing, 100871 China; 3 QingHai Salt Lake Industry Co., Ltd., Qinghai, 816000 China

**Keywords:** Limnology, Chemistry, Mathematics and computing

## Abstract

The output and grade of liquid potash minerals in Qarhan Salt Lake are decreasing year by year, which has become the main problem restricting the sustainable production of potassium fertilizer. The exploitation and utilization of low-grade solid potash ore, which is in the strata of the Qarhan Salt Lake, represents the fundamental framework for the sustainable development of Qarhan Salt Lake’s potash fertilizer. PHREEQC is a simulation software for hydrogeochemistry. In this paper, PHREEQC was applied to simulate temperature, pH value and solvent chemical characteristics which affect the dissolution process of low-grade solid potash minerals. The simulation results indicate that the optimum temperature for ore dissolution is around 25 °C, because, around this temperature, the dissolving ability of solvents to low-grade solid potash minerals is enhanced, while the dissolving ability to halite remains basically unchanged, which is conducive to selective dissolution of low-grade solid potash. It is recommended the temperature is between 20 and 30 ℃. The simulation results show that, when the pH value of solvents is more than 9, although it is advantageous to selective dissolution of low-grade solid potash minerals, the solvent becomes strong alkali solution, which will cause environmental pollution and seriously corrode materials and equipment in actual production, so it is recommended the pH value of the solvent is adjusted between 6 and 8. The simulation results show that, when the values of K^+^, Na^+^, Mg^2+^, Ca^2+^, Cl^−^ and SO_4_^2−^ in the solvent are 0.1%, 2.9%, 3.77%, 0.05%, 15.72% and 0.13% respectively, the solubility of low-grade solid potash ores is stronger, which is more conducive to selective ore dissolution. It is suggested that in actual production, the chemical composition of solvents prepared with old brine and fresh water should be as close as possible to the above chemical composition characteristics.

## Introduction

Qarhan Salt Lake is the largest potash fertilizer production base in China with proven liquid potash mineral resources of 2.44 × 10^8^ tons and solid potash mineral resources of 2.96 × 10^8^ tons^1^. It is difficult to develop these solid potash minerals by traditional mining method of solid potash ore because of its scattered distribution, thin ore bed and low-grade. The solid potash minerals do not meet the formal and official requirements of industrial development, so are called low-grade solid potash ore (abbreviation LGSP ore, afterwards used often). For a long time, the main mining resource is liquid potash minerals in Qarhan Salt Lake. With the extension of mining time of liquid potash, the quantity and quality of iquid potash minerals decreased year by year, which became the main problem restricting the sustainable production of potash fertilizer^2–5^. As a results, the mining of LGSP ore through liquefaction becomes an important and feasible method to meet the sustainable production of potash fertilizer in Qarhan Salt Lake^6^.

Since 1990s, there have been some scholars or research institutes to study the development and utilization of LGSP ore. Qinghai Salt Lake Exploration and Development Institute^7^ studied the recoverable reserves of KCl in Qarhan Salt Lake and demonstrated the feasibility of LGSP ore dissolved by dilute brine. They mainly studied the relationship between time and dissolution rate of LGSP ore. Sun Dapeng and others^8^ proved that low-grade solid carnallite dissolved in the first mining area of Qarhan Salt Lake due to the continuous supply of low concentration brine in the periphery. Hao Aibing^9^ carried out a series of laboratory experiments on LGSP ore dissolution, and pointed out the importance of the concentration and composition of the solvent to the dissolution process, but he mainly used the NaCl solvent. Li Wenpeng and others^10,11^ carried out a numerical simulation study on the LGSP to simulate the dissolution process, and obtained the conclusion that the ion curve in the dissolution driving process is wave like. An Lianying et al.^12^ mainly studied the conversion rate and conversion speed were affected by the difference of potassium concentration in solid and liquid potash ore. Wang Wenxiang et al.^13,14^ studied and discussed the change of hydrodynamic field and hydrochemical field in the process of the solid–liquid transformation of LGSP ore. In 2006, the Institute of Mineral Resources, Chinese Academy of Geological Sciences and QingHai Salt Lake Industry Co., Ltd., undertook the National High Technology Research and Development Program of China (863 Program), namely, “Key Technologies for Liquefaction and Exploitation of LGSP Minerals in Qaidam Basin”, taking the hard to mine LGSP ore in Qarhan Salt Lake as the research object, the LGSP ore mining technology was studied; the main research achievements include finding out the new combination characteristics and distribution rules of potash minerals in the potassium-bearing strata, and modifying the LGSP ore dissolving driving model^15^. Liu Dongqu and others^16^ simulated the dissolving effect of Mg2+ based solvent of the LGSP ore in Qarhan Salt Lake. Wang Xingfu and others^17^ pointed out that the dissolution conversion rate of LGSP ore increased with the increase of LGSP’s grade. Li Xinmeng and others^18^ studied the relationship between the particle size and potassium content of LGSP minerals, and the results showed that the particle size of LGSP minerals decreases gradually, the content of potassium in minerals decreases. Wang Luohai et al.^19^ discussed the impact of “solid to liquid” technology of LGSP on resources and environment, indicating that “solid to liquid” technology has greatly increased the recoverable reserves of potassium resources, extended the service life of Qarhan Salt Lake, improved the utilization rate of Salt Lake resources, and realized the green development of Salt Lake resources. To sum up, although predecessors have made some important understandings and drawn some important conclusions, due to the complexity of hydrological and physicochemical conditions in the process of solid–liquid transformation, and Salt Lakes are always dynamic change, the dissolved mechanism of LGSP ore is still not completely clear.

The dissolution process of LGSP ore in Qarhan Salt Lake is a physical and chemical reaction process of solvent and potash minerals. This process is affected by many factors such as temperature, pH, characteristics of solvent chemical components, solvent flow rate, salt layer structure and porosity. Until now, the factors of temperature and pH value on LGSP ore’s dissolution have not been studied and discussed and the most suitable solvent composition has not been found. In the past, researchers usually used traditional experimental methods to study the LGSP’ dissolution process, however, it is very complicated to study these variables by traditional experimental methods, which requires a lot of manpower, material and financial resources. The hydrogeochemical simulation technology developed on the basis of thermodynamics provides an effective and easy method and way for the study of such problems. Under the reliable parameters, some tedious experimental work can be simulated by computer in a short time^20,21^. A series of equations of Pitzer theory are used in part of hydrogeochemistry computer simulation software, which can describe the activity, ionic strength, dissolution equilibrium of different phase substance and charge balance of solution in high-salinity waters or brine. PHREEQC is one of the hydrogeochemical simulation software^22^. In this paper, we carried out the method of computer simulation (PHREEQC) and laboratory experiment to explore the influence of temperature, pH value and suitable solvent composition on the dissolution of LGSP minerals, which provides scientific basis for reasonable solution mining scheme.

## Introduction to PHREEQC

PHREEQC, which evolved from the Fortran program PHREEQE, is a hydrogeochemical software developed by the U. S. Geological Survey^22,23^. PHREEQC version 3, which is the latest version, is written in the C and C++ programming languages that is designed to perform a wide variety of aqueous geochemical calculations. PHREEQC uses the following several aqueous solution models: The Lawrence Livermore National Laboratory model and WATEQ4F which are belong to two ion-association aqueous models, the Pitzer model and the SIT aqueous model. By using any of these aqueous models, PHREEQC can solve almost all equilibrium thermodynamics and chemical kinetics problems in the interaction system of water, gas, rock and soil, including water solute coordination, adsorption–desorption, ion exchange, surface coordination, dissolution–precipitation and oxidation–reduction^24^. In addition, because of the application of Pitzer model, PHREEQC can simulate the high concentration electrolyte. PHREEQC can be carried out speciation and saturation-index calculations, one-dimensional transport calculations and batch-reaction, inverse modeling and so on^24^.

For multi solute electrolyte solutions, PHREEQC uses a series of equations to describe the activity of water, ionic strength, dissolution equilibrium of different phases, solution charge balance, element composition balance, mass conservation of adsorbent surface and so on. According to the user’s input command, PHREEQC will select some of the equations to describe the corresponding chemical reaction process. For example, the improved Newton–Raphson method is used for iterative solution; the Runge–Kutta method is used for PHREEQC to simulate the dynamic reaction process by integrating the reaction speed in time; for the one-dimensional convection dispersion process of multi-component chemical reaction, PHREEQC uses the split operation technique to calculate the chemical reaction terms count^25^.

Saturation indices (SI) is one of the most widely used indicators in hydrogeochemical research. It studies the saturation state of minerals in aqueous solutions. The SI of minerals in aqueous solution is defined as: *SI* = lg*IAP* *−* lg*K*_*sp*_, where IAP is the activity product of related ions in mineral dissolution solution; K_*sp*_ is equilibrium constant of mineral dissolution reaction at a certain temperature. When SI = 0, the mineral is in equilibrium in the aqueous solution; When SI < 0, it means that the mineral is not saturated in the aqueous solution, and the mineral will be dissolved; when SI > 0, it indicates that the mineral is in the supersaturated state in the aqueous solution, and the mineral will precipitate^26^.

## Research methods

First, using PHREEQC program simulates the most suitable temperature and pH value for the dissolution of LGSP minerals. Secondly, the simulation of solvent composition is carried out under the suitable temperature and pH value conditions. Finally, the laboratory experiment is set to verify the simulation results.

## Simulation analysis by PHREEQC

The possible mineral phases need to be determined before simulation. The most common salt minerals in Qarhan Salt Lake are halite, polyhalite, carnallite, gypsum and sylvite, and a small amount of calcite and dolomite are occasionally seen. The clastic minerals include quartz, mica, albite and chlorite^13,14,27–31^. The clastic minerals are insoluble minerals, calcite and dolomite are not common minerals, and the amount is very small. Therefore, the main mineral phases of Qarhan Salt Lake are gypsum, halite, polyhalite, carnallite and sylvite.

When simulating, only one factor is a variable and the others should be constant. First of all, the suitable temperature is simulated, so the pH and the solvent composition should be constant. After years of exploration, the solution mixed with old brine and Senie Lake’s water is currently used as the solvent to dissolve LGSP ore^6^. The arithmetic mean values of K^+^, Na^+^, Mg^2+^, Ca^2+^, Cl^−^, SO_4_^2−^ in the solvent were 0.23%, 1.60%, 3.69%, 0.05%, 15.23% and 0.13%, respectively^6^. The arithmetic mean value of pH is 6.5, and the arithmetic mean value of density is 1.184 g/cm^3^@@@^6^.

### Effect of temperature on dissolving ability of solvent

The precipitation and dissolution of minerals in water depend on their solubility to a large extent, and the solubility is greatly affected by temperature. Consequently, there should discuss the influence of temperature on the dissolving ability of solvents, and determine the most suitable dissolution temperature.

In the simulation, set pH = 6.5, density = 1.184 g/cm^3^, pe = 4 (default value), and select pitzer.dat database. The values of K^+^, Na^+^, Mg^2+^, Ca^2+^, Cl^−^, SO_4_^2−^ are 0.23%, 1.60%, 3.69%, 0.05%, 15.23% and 0.13%, respectively.

The average temperature of Qarhan Salt Lake is about 25 °C in summer, the highest temperature in summer is 35.5 °C, the average temperature over the years is about 5.1 °C, the lowest temperature in winter is − 29.7 °C, and the average temperature in winter is about − 15 °C^29^. In addition, a maximum temperature of 40 °C is set for comparative study. Therefore, the simulated temperature is set to − 29.7 °C, − 15 °C, 5.1 °C, 25 °C, 35.5 °C and 40 °C respectively.

### Results and discussion

**Figure 1:**
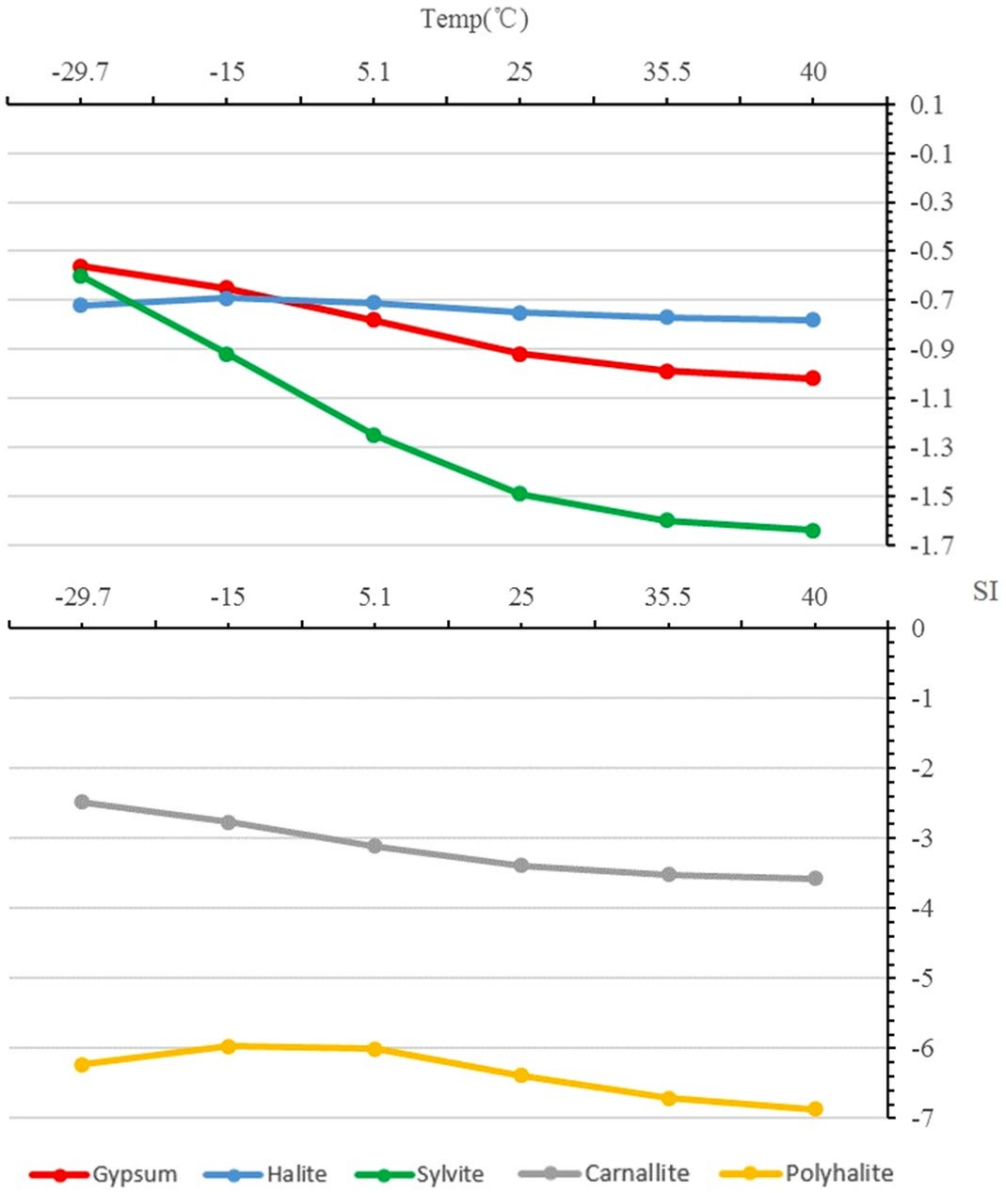
The relationship between SI and temperature.

The simulation results are shown in Fig. 1.

As shown in Fig. 1, taking 25 °C as the demarcation point, when the temperature decreases from 25 to − 29.7 °C, the SI of gypsum, carnallite and sylvite increases linearly with the decrease of temperature, which indicates that the dissolving ability of solvent to gypsum, carnallite and sylvite decreases with the decrease of temperature. When the temperature increases from 25 to 40 °C, the SI of gypsum, carnallite and sylvite decreases with the increase of temperature, but the decrease rate tends to be flat, which indicates that the solubility of gypsum, carnallite and sylvite increase with the increase of temperature, however, the increase is gradually to be weaker. The change for the SI curve of halite and polyhalite is relatively small, which indicates that the dissolving ability of solvent to halite and polyhalite is basically unchanged with the change of temperature. On the whole, the SI of polyhalite is the smallest, that is to say, the solvent has the strongest solubility to polyhalite. However, due to the formation of gypsum in the process of dissolution of polyhalite, in the case of no hydrodynamic or weak hydrodynamic condition, the polyhalite is easy to be wrapped by the formed gypsum, resulting in the stagnation of the dissolution of polyhalite^32^.

Above all, in the cold season with relatively low temperature, the solubility of solvent to carnallite and sylvite decreases, however, due to the weak effect of temperature on the dissolution ability of halite, the halite continues to dissolve in the cold season, which is not conducive to LGSP ore dissolution. However, some enterprises inject solvents into the field to dissolve LGSP minerals in winter. When the temperature is higher than 40 °C, although the dissolving capacity of the solvent to potash minerals increases, the increase is not very obvious, moreover, the higher the temperature is, the stronger the degree of evaporation will be, which is not conducive to ore dissolution. Consequently, the temperature that is around 25 °C is most suitable for LGSP dissolution. We recommend that the temperature range of LGSP dissolution is between 20 and 30 °C.

Qarhan Salt Lake is located in the Qinghai-Tibet Plateau, where the humid and hot air from South Asia is blocked by the Himalayas, so it cannot meet the cold air from Siberia, therefore, it cannot form precipitation conditions^33^. The climate in Qarhan Salt Lake belongs to the typical continental arid climate, which is windy and dry all the year round^33^. According to the meteorological data statistics collected by Qinghai Salt Lake Industry Co., the temperature of Qarhan Salt Lake from May to October is between 20 and 30 °C. In terms of actual production, selecting May to October with relatively high temperature is conducive to the dissolution of LGSP minerals.

### Effect of pH on dissolving ability of solvent

During the pH simulation, it can be seen from the above simulation that the temperature has better to be set at 25 °C. The density is 1.184 g/cm^3^, pe is 4 (default value), and select pitzer.dat database. The values of K^+^, Na^+^, Mg^2+^, Ca^2+^, Cl^−^, SO_4_^2−^ are 0.23%, 1.60%, 3.69%, 0.05%, 15.23% and 0.13%, respectively. The initial pH of the solvent was 6.5. The pH values were adjusted by adding HCl or NaOH to 1.0, 2.0, 3.0, 4.0, 5.0, 6.0, 6.5, 7.0, 8.0, 9.0, 10.0, 11.0 and 12.0, respectively.

#### Results and discussion

**Figure 2:**
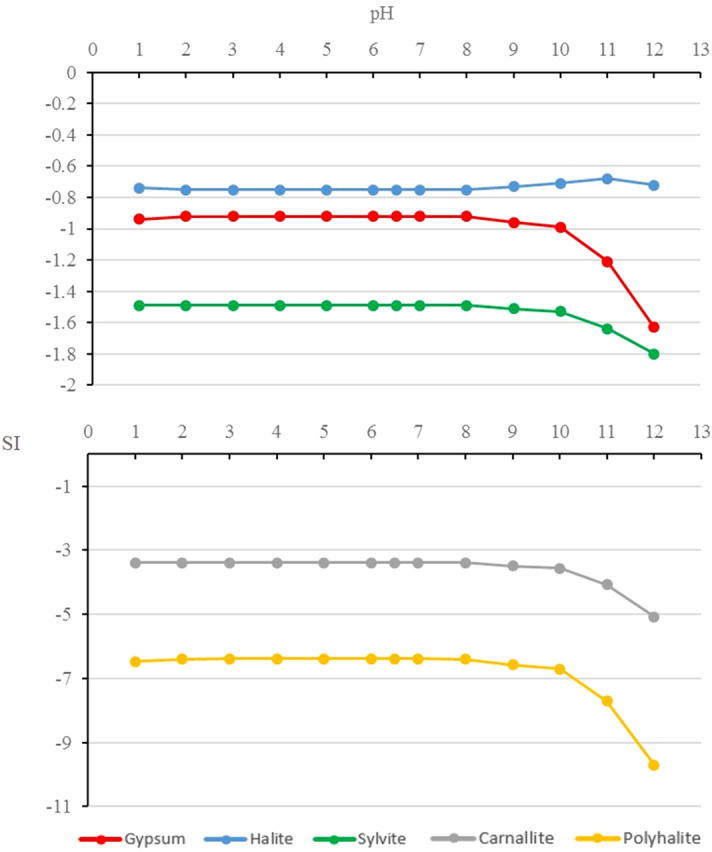
The relationship between SI and pH.

The simulation results are shown in Fig. 2.

**Figure 3:**
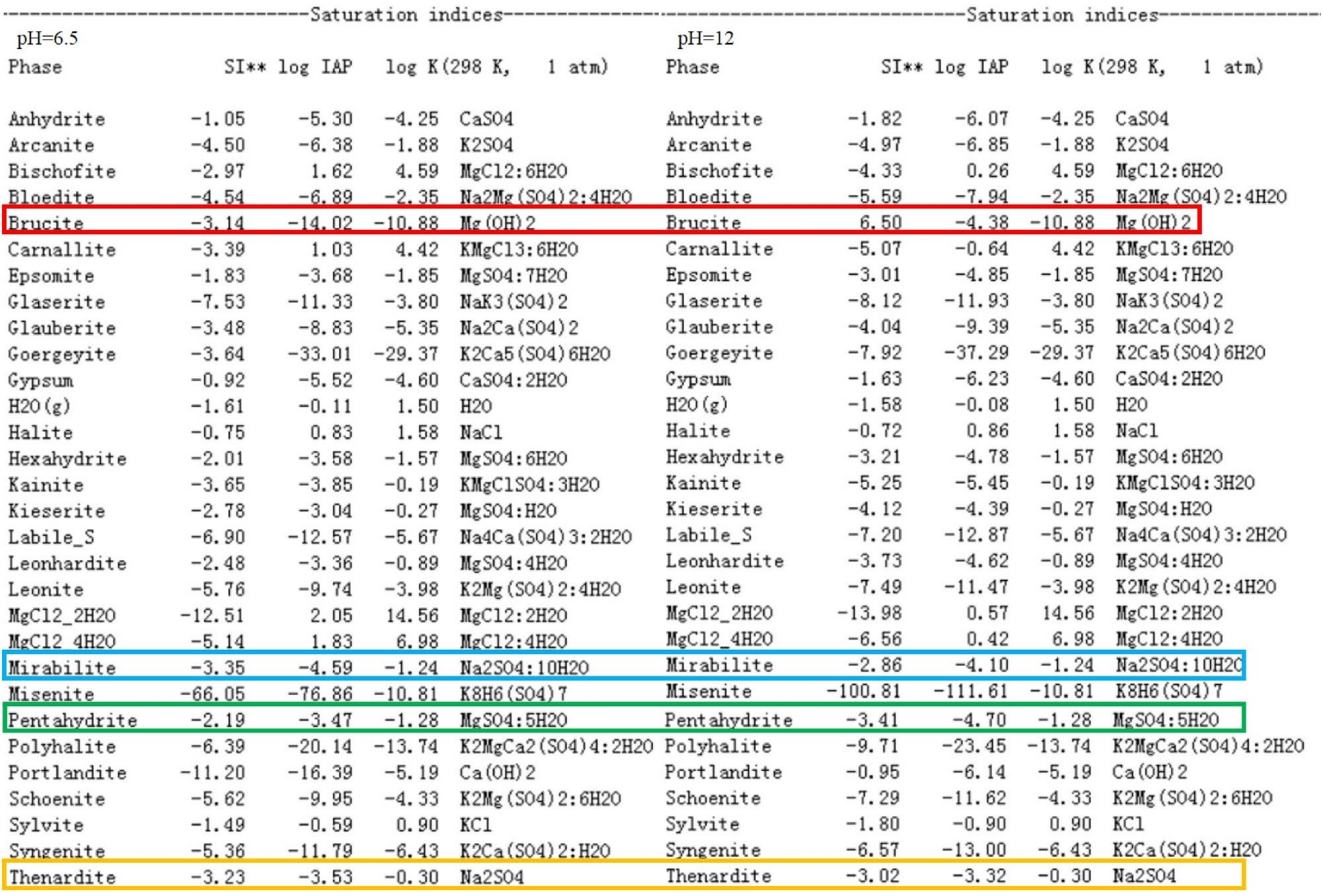
The SI of different minerals under different pH.

The only pH related reaction in the solvent is sulfate, the reaction form as follows: ${\mathrm{SO}}_{4}^{2-}+{\mathrm{H}}^{+}={\mathrm{HSO}}_{4}^{-}$. When pH is about 2, the activities of ${\mathrm{SO}}_{4}^{2-}$ and ${\mathrm{HSO}}_{4}^{-}$ are similar. Only at a lower pH value, the pH of the solvent will affect the activity of sulfate and the mineral saturation index (SI). As shown in Fig. 2, when the pH value of the solvent is gradually reduced from 6.5 to 1.0, the mineral saturation index (SI) of gypsum, halite, polyhalite, carnallite and sylvite is basically unchanged, which indicates that in the process of solvent acidification, the mineral dissolving capacity of the solvent to gypsum, halite, polyhalite, carnallite and sylvite is basically unchanged. When the pH value of solvent increases from 6.5 to 12, especially after pH > 10, the mineral saturation index (SI) of gypsum, polyhalite, carnallite and sylvite graduaaly decreases, and the mineral saturation index (SI) of halite increases, which indicates that in the process of solvent alkalization, especially when the solvent is strongly alkaline solution, the dissolving ability of solvent to gypsum, polyhalite, carnallite and sylvite is enhanced, and the dissolving ability of solvent to halite is weakened. Why do these phenomena appear? It is mainly caused by the following reasons: chloride or sulfate minerals containing calcium and magnesium will dissolve in strong alkaline solutions and precipitate in the form of portlandite (Ca(OH)_2_) and brucite (Mg(OH)_2_). Taking polyhalite (K_2_MgCa_2_(SO_4_)_4_·2H_2_O) as an example, the following reaction will occur in strong alkaline solution: K_2_MgCa_2_(SO_4_)_4_·2H_2_O + 2NaOH = 2CaSO_4_↓ + Mg(OH)_2_↓ + 2 K^+^ + 2Na^+^  + 2·${\mathrm{SO}}_{4}^{2-}$ + 2H_2_O. Therefore, with eh increase of the alkalinity of the solvent, the dissolving ability of the solvent to LGSP ore will be enhanced. In addition, due to the increase of Na^+^ in the solution, the same ion effect leads to the increase of mineral saturation index of halite, thus the dissolving ability of the solvent to halite decrease. The mineral saturation index of other Na^+^—bearing minerals will also increase, as shown in Fig. 3.

It can be seen from the above when the pH value decreases from 6.5 to 1, the dissolving ability of the solvent to LGSP minerals remains basically unchanged. When the pH value increase from 6.5 to 12, especially from 9 to 12, when the solvent shows strong alkalinity, the dissolving ability for LGSP minerals is strengthened. Although the dissolving ability for LGSP minerals is enhanced, because the strong alkaline solution pollutes the environment and seriously corrodes materials and equipment in actual production, the strong alkaline solvent is not suitable for LGSP dissolution. Therefore, the pH value of solvent maintained around 6.5 is suitable for LGSP minerals dissolution. In terms of actual production, we recommend the pH value of solvent is adjusted between 6 and 8.

### Effect of solvent chemical characteristics on dissolving ability of solvent

A suitable solvent is very important for the dissolving of LGSP ore. If the concentration of the solvent is too low or fresh water is used directly, a large amount of surrounding rock, mainly halite, will be dissolved, which will cause the collapse of salt bed. The collapse of salt bed will affect the permeability and porosity of the strata, and also form geological disasters and threaten the personal safety of field works. If the concentration of solvent is too high, salt minerals will be easy to crystallize and precipitate, which will block the pore passageway and affect the porosity, thus affecting the permeability of salt bed. The ideal solvent is to dissolve the LGSP ore selectively, but try not to dissolve other minerals such as halite. Therefore, it is very important to select a suitable solvent for the mining of LGSP ore.

Is the previous solvent (defined as the initial solvent) used in the dissolution of LGSP minerals in Qarhan Salt Lake the best suitable solvent? It can be used PHREEQC program to simulate whether the initial solvent is the most fitful. Based on the above simulation, set the temperature = 25 °C, pH = 6.5, pe = 4 (default value), and select pitzer.dat database. Only change the chemical composition of the solvent to simulate. Through dozens of simulations with PHREEQC program, it was found that when the concentration of K^+^, Na^+^, Mg^2+^, Ca^2+^, Cl^−^ and SO_4_^2−^ of the solvent (defined as simulated solvent) are 0.1%, 2.9%, 3.77%, 0.05%, 15.72% and 0.13%, respectively (see Table 1), the solubility of LGSP minerals is stronger (see Table 2 for details).

**Table 1:** The chemical characteristics of solvents (unite: %).

Ions	K^+^	Na^+^	Mg^2+^	Ca^2+^	Cl^-^	SO_4_^2-^
Initial solvent	0.23	1.60	3.69	0.05	15.23	0.13
Simulated solvent	0.1	2.9	3.77	0.05	15.72	0.13

**Table 2:** The different SI in different solvents.

Mineral phase	Carnallite	Gypsun	Halite	Polyhalite	Sylvite
Initial solvent-SI	− 3.39	− 0.92	− 0.75	− 6.39	− 1.49
Simulated solvent-SI	− 3.55	− 0.87	− 0.38	− 6.94	− 1.78

It can be seen from Table 2 that the mineral saturation index (SI) of the simulated solvent for halite and gypsum increases, while the mineral saturation index (SI) for carnallite, polyhalite and sylvite decreases. Compared with the initial solvent, the dissolving ability of the simulated solvent to halite and gypsum is weakened, while to carnallite, polyhalite and sylvite is enhanced, which is conducive to LGSP dissolution. In addition, in the ore-bearing layer of Qarhan Salt Lake, potash minerals often occur between the pores of halite crystal particles in the form of disseminated^4,34–37^. The simulated solvent can dissolve a small amount of halite, which can release the potash minerals between the pores of halite crystal particles into the solution. Because it does not cause a large amount of halite to dissolve, it will not cause salt layer collapse. To sum up, the simulated solvent may be more suitable for LGSP dissolution, which can be verified by the following laboratory experiments.

#### Laboratory experiments

##### Experimental conditions and methods

Experimental equipment and reagent: beaker, measuring cylinder, transfer pipe, volumetric flask, incubator, ultrapure water and reagents (analytical reagent).

Analytical method: ICP-OES.

Experimental ore sample: the ore samples used in the experiment were collected from Qarhan Salt Lake and were from the same ore-bearing layer. The ore samples were mixed evenly, then they were divided into two equal parts.

Experimental steps:

- According to the Table 1, 2 L initial solvent and simulated solvent were prepared respectively, and the pH of the two solvent were adjusted to 6.5. Since the concentration of Ca^2+^ and SO_4_^2−^ were pretty low in the initial solvent and simulated solvent, meanwhile, gypsum is difficult to soluble in solutions. Consequently, Ca^2+^ and SO_4_^2−^ in either the initial solvent or simulated solvent were not added.
- Two parts of ore samples with the same mass (850 g each) were weighed and put into two 2 L beakers respectively. The prepared initial solvent and simulated solvent (1 L each) were poured into two beakers respectively. The two beakers were placed in a constant temperature incubator at 25 °C. The liquid phase samples were taken from two beakers respectively every 1 h, and 5 mL each time. The experiment lasted 8 h, and 8 liquid samples were taken from each beaker, 16 liquid samples in total.
- Carry out chemical analysis on the 16 liquid samples.
- Data analysis.

##### Experimental results and discussion

**Figure 4:**
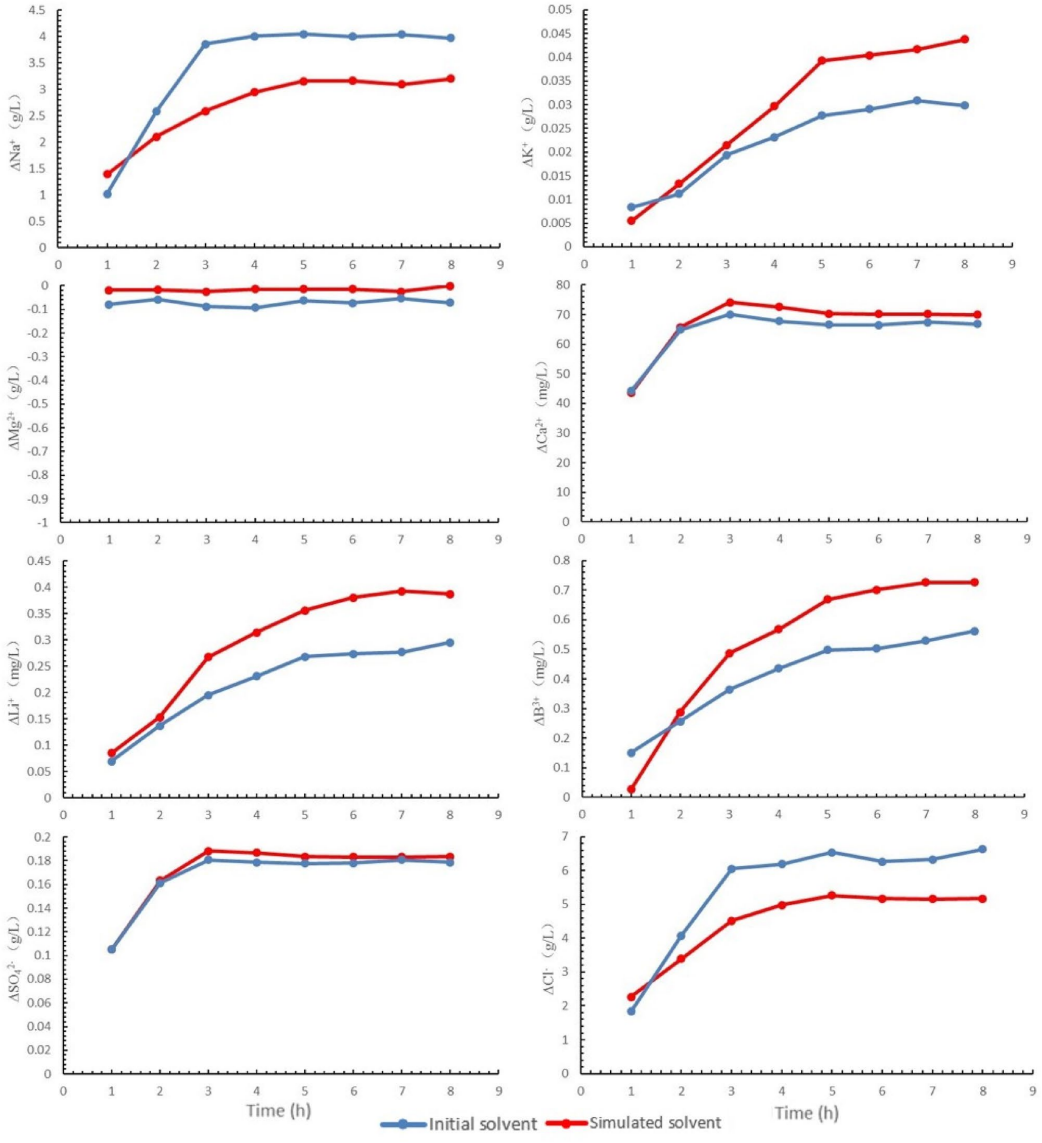
Ion change in the process of minerals dissolution.

Figure 4 shows the experimental results of the dissolving ore samples of the two solvent (initial solvent and simulated solvent) respectively, and “∆” represents the net change.

As shown in Fig. 4, the change trend of Na^+^ and Cl^−^ is basically the same. Compared with the initial solvent, the net increase of Na^+^ and Cl^−^ is relatively small when use the simulated solvent, so the solubility of Halite by the simulated solvent is low, which is good to the salt bed, for not cause the collapse of the salt layer. Compared with the initial solvent, the net increase of K^+^ is relatively high and increases relatively quickly when use the simulated solvent. In the process of ore dissolution by the two solvents, the change of Mg^2+^ is small. In the process of preparing of the two solvents, Ca^2+^ and SO_4_^2−^ were not added. But after 8 h of ore dissolving, Ca^2+^ and SO_4_^2−^ were detected in the two solutions, and their change trends were basically the same, indicating that gypsum or polyhalite were dissolved. In addition, compared with the initial solution, the net increase of Ca^2+^ and SO_4_^2−^ was relatively higher when the simulated solvent was used to dissolve the ore samples. Li^+^ and B^3+^ were also not added during the preparation of the two solvents, however, a small amount of Li^+^ and B^3+^ were detected in the solution after 8 h of ore dissolution, indicating that there was a small amount of Li^+^ and B^3+^—bearing minerals dissolved.

From the above analysis, compared with the initial solvent, the simulated solvent is more favorable for selective dissolution of LGSP minerals. Therefore, in the actual production, the chemical composition of the solvent prepared with old brine and Senie Lake’ water should be as close as possible to the simulated solvent.

## Conclusions

In this paper, through the research method by combining the PHREEQC program simulation and laboratory experiments, the main conclusions are drawn as follows:

1. In the dissolving process of LGSP minerals in Qarhan Salt Lake, the temperature should be around 25 °C, and it be recommended the temperature is between 20 and 30 °C, which is the most suitable for dissolving ore. Around this temperature, the dissolving ability of the solvent to LGSP minerals is enhanced, and the solubility of halite is basically unchanged, which is conducive to the selective dissolution of LGSP ore. Considering the natural conditions of Qarhan Salt Lake, selecting May to October with relatively high temperature is in favor of the dissolution of LGSP minerals.
2. In the dissolving process of LGSP minerals in Qarhan Salt Lake, when pH is more than 9, the dissolving ability of solvent to gypsum, polyhalite, carnallite and sylvite is strengthened, and the dissolving ability to halite is weakened, which is beneficial to selective dissolution of LGSP ore. However, with the pH value increasing, although the dissolving ability of LGSP minerals is increased, the solvent becomes strong alkaline solution which pollutes the environment and seriously corrodes materials and equipment in actual production. Therefore, in terms of actual production, it be recommended the pH value of solvent is adjusted between 6 and 8.
3. In the dissolving process of LGSP minerals in Qarhan Salt Lake, when the chemical compositions of solvent, K^+^, Na^+^, Mg^2+^, Ca^2+^, Cl^−^ and SO_4_^2−^, are 0.1%, 2.9%, 3.77%, 0.05%, 15.72% and 0.13%, respectively, the dissolving ability of solvent to LGSP minerals is enhanced, which is more favorable to selective dissolution of LGSP minerals. In actual production, the chemical composition of the solvent prepared with old brine and Senie Lake should be as close as possible to the composition showed above.

In order to better study the dissolving process of LGSP minerals, the interaction between temperature, pH and solvent chemical characteristics should be considered. We plan to do the orthogonal simulated experiment in terms of temperature, pH value and solvent chemical characteristics in the future. In addition, we will also do other factors that affect the dissolving process of LGSP minerals, such as porosity of the strata.
